# Revolutionizing solid-state single-photon sources with multifunctional metalenses

**DOI:** 10.1038/s41377-023-01372-3

**Published:** 2024-01-17

**Authors:** Fei Ding

**Affiliations:** Centre for Nano Optics, University of Southern Denmark, Campusvej 55, Odense M, DK-5230 Denmark

**Keywords:** Metamaterials, Quantum optics

## Abstract

Ultrathin multifunctional metalenses are demonstrated to control the multiple degrees of freedom of a single-photon source in hexagonal boron nitride.

Quantum photonics, a pivotal field in the quantum realm, leverages the unique properties of light at the quantum level^1^. Central to this domain are deterministic single-photon sources, which emit individual photons sequentially from spontaneous emission^2^, a cornerstone for quantum communication, computing, and secure encryption. However, the interaction between light and solid-state single-photon emitters (SPEs, such as quantum dots, color centers in diamonds, and defects in two-dimensional materials) under ambient conditions is fundamentally weak and difficult to control. Therefore, the resulting single-photon sources exhibit several issues, such as low collection efficiency, lack of directionality, and poor polarization/phase properties. To create complex quantum light states and make full use of multiple degrees of freedom (DoFs) of single photons, such as polarization and orbital angular momentum, one should set up a complicated optical system with a set of discrete components (polarizers, wave plates, lenses, spatial light modulators, etc.), an approach that is inherently cumbersome suffering from bulky configurations, alignment challenges, instability, losses, and limited functionalities. Optical metasurfaces, extremely thin nanoantennas arranged in a well-considered pattern, have unprecedented capabilities in manipulating all properties of classical and nonclassical light, hereby making a unique promising platform for quantum nanophotonics^3–6^. In particular, they provide a platform for generating and manipulating the quantum states of photons^7–10^, facilitating novel ways to control quantum light for integrated quantum photonic devices.

The paper “Arbitrarily structured quantum emission with multifunctional metalens” published in eLight is a testament to the rapid advancements in quantum photonics^11^. The research team, led by Dr. Chi Li and Dr. Haoran Ren from Monash University, Prof. Junsuk Rho from Pohang University of Science and Technology, and Prof. Igor Aharonovich from the University of Technology Sydney, introduces a novel multifunctional metalens that redefines the control of quantum emission from SPEs in hexagonal boron nitride (hBN) at room temperature. This designed metalens enables simultaneous mapping of quantum emission from ultra-bright defects in hBN and imprinting of an arbitrary wavefront onto orthogonal polarization states of the sources, owing to its capability of molding the directionality, polarization, and OAM DoFs simultaneously. As such, this hybrid quantum metalens system enables simultaneous manipulation of multiple DoFs of a quantum light source, as shown in Fig. 1. In the design, the authors utilized low-loss hydrogenated amorphous silicon as the material to build the metalens unit cells, which has a negligible extinction coefficient in the emission spectrum of hBN SPEs, leading to a reasonably high collection efficiency of 0.3. Capitalizing on the design, three different polarization-splitting metalenses have been fabricated and measured with SPEs to validate their capabilities to control the directionality and polarization of single-photon emission at the same time. Furthermore, the authors implemented more complicated metalens that encode different helical phase fronts (OAM modes) in addition to the directionality and polarization.

**Fig. 1:**
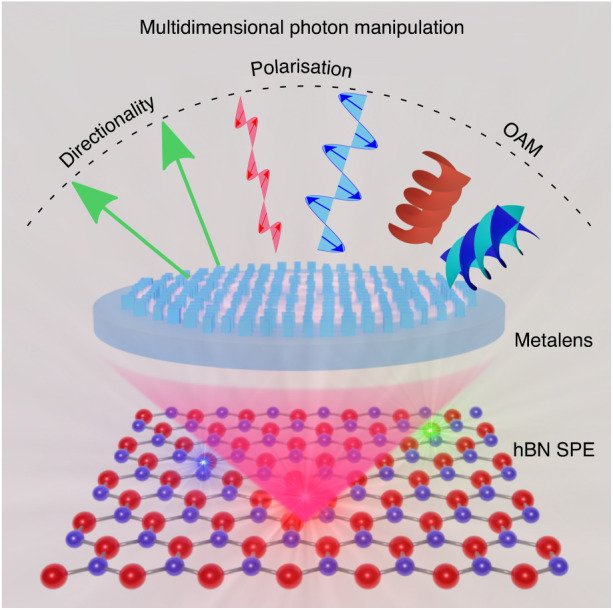
Schematics of multifunctional metalens for manipulating the single-photon emission from hBN SPEs. Reprinted with permission from ref. ^11^

The study showcases the metalens’s ability to manipulate quantum emission from hBN defects, imprinting arbitrary wavefronts onto orthogonal polarization states. The multifunctional nature of these metalenses provides a crucial stepping stone towards advanced quantum computing, secure communication, and enhanced quantum sensing capabilities. We believe that such quantum metasurfaces will be growing rapidly as a unique and enabling platform for generating, routing, and manipulating quantum light states, due to their superior capability to simultaneously control multiple DoFs of photons in an independent and simultaneous fashion. Despite the groundbreaking nature of this research, the multifunctional metalenses that have been employed to manipulate single-photon emission from hBN SPEs are still external components, i.e., separated from the photon sources. Although directly integrating hBN SPEs to metalenses could be possible by the addition of a transparent spacer^8^, adapting device architectures and aligning approaches are nontrivial and need further investigation. Meanwhile, integrated quantum metasurface chips that simultaneously generate photon-state and conduct high-dimensional quantum entanglement are still to be developed. Furthermore, the static nature of the demonstrated quantum metasurfaces so far severely limits the range of available functionalities, calling thus for the development of spatiotemporal quantum metasurfaces with new research avenues and breakthroughs in flat quantum photonics^6,12^.
